# A Workflow for Assessing Particle Counts of Mixed Micro- and Nanoplastics in Exposed Laboratory Animals

**DOI:** 10.3390/nano15110812

**Published:** 2025-05-28

**Authors:** Lauren Gaspar, Sarah Davis, Giuseppe Coppotelli, Andrew J. Davies, Coleen C. Suckling, Jaime M. Ross

**Affiliations:** 1 Department of Biomedical and Pharmaceutical Sciences, College of Pharmacy, University of Rhode Island, Kingston, RI 02881, USA; lgaspar@uri.edu (L.G.); gcoppotelli@uri.edu (G.C.); 2 George and Anne Ryan Institute for Neuroscience, University of Rhode Island, Kingston, RI 02881, USA; 3 College of the Environment and Life Sciences, University of Rhode Island, Kingston, RI 02881, USA; sarahdavis@uri.edu (S.D.); davies@uri.edu (A.J.D.); 4 Graduate School of Oceanography, University of Rhode Island, Narragansett, RI 02882, USA

**Keywords:** microplastics, nanoplastics, methods, detection, tissues, health, pollution

## Abstract

Micro- and nanoplastics (NMPs) have recently gained attention as one of the most pervasive and potentially dangerous environmental pollutants. An increasing number of studies have explored the presence and potential health consequences of NMPs exposure, leading to calls for techniques to assess their bioaccumulation. Significant gaps that remain in this growing field of research are methodologies to quantify discrete particle counts of NMPs below 5 μm in size and evaluate the recovery rate of such methods to ensure accuracy and comparability across studies. To address these gaps, we aimed to develop a high-throughput protocol for the extraction, isolation, and quantification of a 1:1 volume mixture of 2 and 0.1 μm pristine fluorescently labeled spherical polystyrene NMPs (PS-NMPs) from mouse tissue, and to determine recovery rates of both sizes to assess the success of the methodology. We found that we were able to successfully recover 85% of 2 μm NMPs and 30% of 0.1 μm NMPs, and that this workflow could be applied to tissues from mice experimentally exposed to a concentration gradient of PS-NMPs to detect differences in accumulation. This methodology is the first to attempt a continuous workflow to assess particle counts of both micro- and nanoplastics from biological tissues, including calculations of recovery rates, and we anticipate that the workflow described here may be applied and modified in future studies to answer outstanding questions regarding the accumulation of small NMPs that may not be addressed with traditional techniques. Additionally, in identifying the significant differences in recovery rates for micro- versus nanoplastics, we highlight the considerations that must be taken into account for nanoplastics that are often not discussed within the NMPs literature.

## 1. Introduction

Over the past 20 years, global plastic production has more than doubled, exceeding 400 million tons annually, and is projected to double again by 2050 [1]. The increase in production has also led to a sharp rise in plastic pollution, with around 80% of plastic waste accumulating in the natural environment [2]. Once in the environment, plastics do not biodegrade but rather fragment into micro- (<5 mm) and nano- (≤0.1 μm) plastics (NMPs) [3,4]. NMPs have gained significant attention in recent years, as their small size, light weight, and high surface area make them one of the most transportable and easily ingested pollutants. Recent studies have detected NMPs in remote corners of the world [5,6], in various wildlife species [7,8], and most recently in human tissues [9,10,11,12,13], where they have been shown to have adverse health effects including increasing inflammation [14,15], dysregulating gut microbiota [16,17], and altering reproductive function [18,19]. These findings have driven the need for more research to better understand the potential impacts of NMPs across a variety of scientific disciplines.

Current NMPs research includes environmental sampling of water [20,21,22], soil [23,24,25], plants [26,27], wildlife [28,29,30], processed food products [31,32], and even human tissues [9,10,11,12,13] to assess global NMPs accumulation. Additionally, numerous studies have been dedicated to exploring the health impacts of NMPs exposure through the use of both marine [33,34] and mammalian [35,36] models. These studies, however, all face numerous challenges in assessing NMPs accumulation as a result of widely varying NMP particle characteristics and environmental conditions that can impact NMPs transport/accumulation. The fate of NMPs in the environment may be influenced by their particle characteristics, including shape, size, polymer type, bio-fouling status, chemical additives, and adsorbed pollutants, as well as environmental conditions such as location, season, weather patterns, human activity, and sampling methods [37,38]. While it is very difficult for a single study to examine and consider all of these factors, there are certain trends that begin to emerge across studies that are largely directing the field of NMPs research [39]. One such trend is the recent focus on smaller NMPs (≤5 μm), which are estimated to make up as high as 90% of all NMPs [40], but are lacking in laboratory studies as they may evade capture during sample collection or are too small for detection. These particles, however, are critical to assess since they have been identified as being potentially the most abundant [41,42] and toxic size class of NMPs, and their exclusion from analyses may lead to severe underestimation of actual NMPs accumulation and toxicity [43]. NMPs in this size range have been shown to be highly permeable across biological membranes, and their high surface area may allow for greater interaction with biological systems, thus increasing toxicity [44,45].

In the previous study by Gaspar et al. [46], we explored the effects of acute (3 weeks) exposure to a 1:1 volume mixture of 2 and 0.1 μm pristine fluorescently labeled polystyrene NMPs (PS-NMPs) via drinking water on overall health in C57BL/6J mice, the most widely used mouse strain as a proxy to studying human conditions [47]. Although 2 μm falls into the standard “microplastic” size class and 0.1 μm falls closer to the definition of “nanoplastic”, for simplicity’s sake and uniformity we will herein refer to both sizes as “NMPs”. The goal of the previous study was to understand the potential impacts of smaller NMPs exposure through ingestion on mammalian health. This study in particular was one of the first to investigate consumption of NMPs ≤ 5 μm in size, which presented significant challenges in addressing the bioaccumulation of NMPs, as their small size put them below the limit of detection/resolution for many standard techniques.

Since techniques to analyze smaller NMPs are limited, we initially used widefield fluorescence microscopy to visualize the accumulation of PS-NMPs in cryosectioned tissues from exposed mice [46]. Using this approach, larger 2 μm PS-NMPs in the tissues could be identified, but it was extremely labor intensive. Additionally, tissue autofluorescence was found to be a confounding factor that would mask NMPs fluorescence and prevented detection of the smaller 0.1 μm PS-NMPs. In searching the literature, it was found that many studies investigating NMPs ≤ 5 μm either did not report measurements of NMPs accumulation [48,49,50] or used measures of total fluorescence intensity (i.e., microplate readers [51,52] or fluorescence intensity of total tissue sections [53]) to assess accumulation. While these techniques can be useful in providing a broad overview of NMPs transport and accumulation, they cannot determine the actual number of particles within tissue, which is critical to more accurately understand how NMPs travel throughout the body. Quantitative particle counts are also needed to identify potential differences in NMPs burden between study groups (i.e., healthy versus disease-state model) that may not be distinguishable with a fluorescence intensity or mass-based measurement. Additionally, we found that studies using similar sizes of NMPs have rarely attempted to determine a recovery rate for their extraction methods or include positive controls, which makes it extremely difficult to determine how reflective reported values are of true NMPs accumulation [54,55]. Of the recovery rate reports that do exist for NMPs, they may range from <5% to nearly 100% [56,57,58], but it is imperative to note that these values will be heavily impacted by the size of NMP, the sample matrix, and whether the recovery rate is based on mass or particle count. To date, no studies have provided a recovery rate based on particle count for NMPs ≤ 5 μm from tissues which has made assessing their true biological accumulation nearly impossible. Given the increased focus on and potential health hazards associated with this size class of NMPs, the development of a more suitable method to evaluate their presence in exposed tissues was warranted.

For larger NMPs, the primary methods that have been used by previous studies are mass-based, spectroscopy-based, and microscopy-based techniques. Mass-based methods, such as pyrolysis–gas chromatography/mass spectrometry (Py-GC/MS), have many advantages in NMPs research, including relatively simple sample preparation and a detailed chemical analysis of particles [59]; however, these techniques cannot provide information on particle counts and may not be ideal for smaller NMPs, which have a very low mass. This can make it difficult to distinguish the NMPs from background spectra and to differentiate concentration differences between samples, as large changes in particle count may correspond to very minute changes in mass, challenges which also arise with spectroscopy-based methods [60]. Techniques such as Raman spectroscopy and Fourier transform infrared spectroscopy (FTIR) can be used to quantify and determine the chemical composition of NMPs, but may not be compatible with NMPs smaller than 1 μm due to poor signal to noise ratios [61,62] and can be very time consuming, taking hours to analyze a single NMP and often requiring the sub-sampling of tissues [63]. Sub-sampling can pose an issue in NMPs processing, as it may not accurately reflect total NMP accumulation due to the inhomogeneous distribution of particles within a sample [64]. These techniques have also been traditionally used with sample matrices such as water [65,66], but face challenges with matrices such as biological tissue, which may contain endogenous compounds (such as amide groups at 1200–1600 cm^−1^ and components of carboxylic acids at around 1700 and 2500–3000 cm^−1^) that can mask NMP spectrums [64,67]. In recent years, there has been an increased need to develop methods for quantifying sub-micron NMPs, including the use of techniques such as scanning electron microscopy (SEM) [68,69] or atomic force microscopy (AFM) [70,71], for example. While these techniques can be very powerful and allow for resolution at the nano-scale, they can also be extremely time consuming and expensive. This often reduces the number of samples that can be realistically included in a study or will require sub-sampling of tissues.

While each of these aforementioned techniques have been ground-breaking in driving forward NMPs research as a whole, there are still significant improvements that must be made. To date, none of these methodologies have been able to achieve particle counts for NMPs smaller than 5 μm from whole biological tissues and validate the percent recovery of these methods, which has created a significant gap in our understanding of NMPs of this size and what their true bioaccumulation may be. To attempt to address this question, we aimed to employ the recent advances in spinning-disk confocal microscopy to allow for relatively high-throughput quantification of NMPs smaller than 1 μm. Spinning-disk confocal microscopes feature multiple pinholes which allow for rapid scanning of samples while maintaining the resolution of traditional confocal microscopes. This can be particularly advantageous, as many of the observed toxic effects of microplastics have been attributed to interactions of individual particles with biological systems, so providing an estimation of particle count as opposed to the total mass of NMPs may provide a greater understanding of their toxicity [72,73].

The overall aim of this study was to help advance the field of NMPs exposure research in health sciences by developing a workflow for detecting and quantifying particle counts for small NMPs, which may enhance our understanding of their impacts, bioaccumulation, and clearance. To achieve this, we developed methods to extract, isolate, and quantify 2 and 0.1 μm polystyrene NMPs from mouse tissue and measured success by evaluating the recovery rate of our methodology. These approaches were then applied to a proof-of-concept trial in which kidney tissue, collected from our previous laboratory study [46] where mice were exposed to a concentration gradient of PS-NMPs via drinking water, was evaluated for PS-NMPs bioaccumulation. The goal of developing this workflow is that it may be modified and applied in future NMPs exposure studies for the quantification of individual NMPs < 2 μm in whole tissue samples, which has not previously been achievable, and is essential to understand NMPs transport and toxicity.

## 2. Results

### 2.1. Recovery Rate Calculations

**Figure 1:**
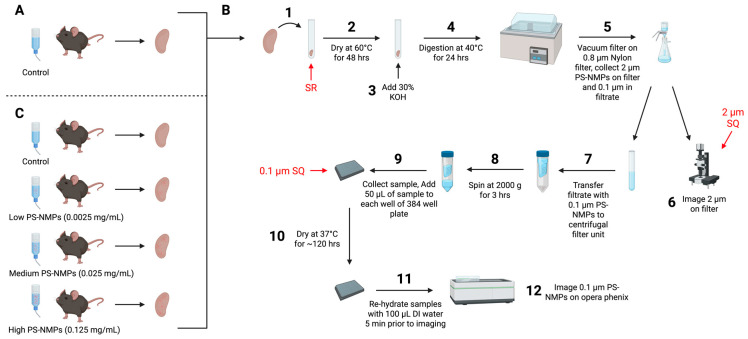
**Flow chart for the extraction, isolation, and quantification of PS-NMPs from tissue.** Steps outlining the methods for extracting 2 and 0.1 μm fluorescent PS-NMPs from (**A**) mouse tissue used for recovery rate calculations (N = 45), followed by (**B**) general workflow and then a (**C**) proof-of-concept study (N = 20, 5 per group). Points of spiking during the workflow for the recovery rate calculations are shown in red. Spike recoveries (SR) were introduced at the start of the workflow and undergo the entire extraction process to mimic potential recovery from actual samples. Spike quantifications (SQ) are spiked immediately prior to imaging to quantify and confirm PS-NMPs present in the initial spiking volume. The schematic was created with BioRender.com (accessed on 27 September 2023).

**Figure 2:**
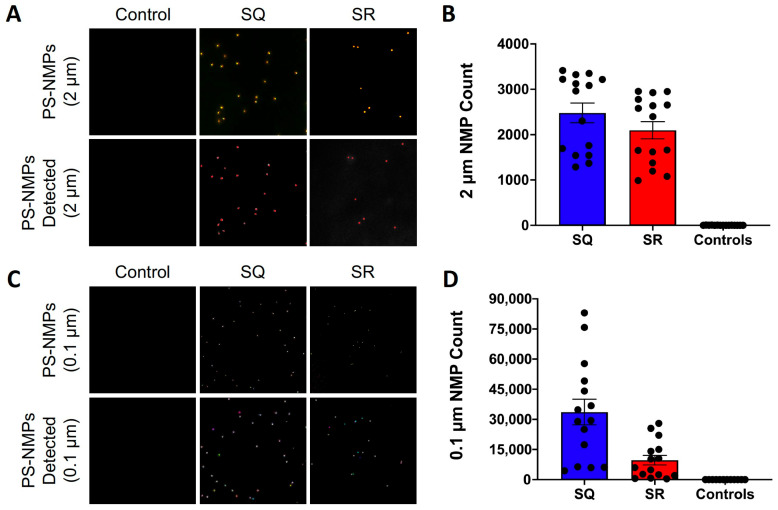
**PS-NMPs recovery rate calculations.** (**A**) Representative fluorescence images of 2 μm PS-NMPs acquired using an automated widefield fluorescence microscope (Olympus BX63) under 10× magnification. SR and SQ samples were automatically counted using appropriate software (CellSens Dimension^TM^ Count and Measure feature). Images shown are before software recognition (**upper panels**) and after software recognition (**lower panels**) to demonstrate accuracy. Field of view = 250,000 μm^2^. (**B**) Quantification of 2 μm PS-NMPs for SQ samples (N = 15), SR samples (N = 15), and control samples (N = 15). (**C**) Representative fluorescence images of 0.1 μm PS-NMPs acquired using an automated spinning-disk confocal for high content screening (Opera Phenix) and automatically counted using appropriate software (Harmony). Images shown are before (**upper panels**) and after (**lower panels**) spot detection to demonstrate the accuracy of automatic counting. Field of view = 10,000 μm^2^. (**D**) Quantification of 0.1 μm PS-NMPs for SQ samples (N = 15), SR samples (N = 15), and control samples (N = 15).

To assess the recovery rate of our protocol, samples were spiked and processed as described in the Materials and Methods section and within Figure 1, and the mean ± SEM were calculated for each group. The average count for the 2 μm PS-NMPs was 2477 ± 219 for the SQ samples, while the average count for the SR samples was 2094 ± 193, giving an estimated recovery of approximately 85%. Control samples were found to contain minimal NMPs contamination with an average count of ~2 ± 1 (Figure 2A,B). For the 0.1 μm PS-NMPs, the average count of the SQ samples was 33,622 ± 6426, while the average count of the SR samples was 9655 ± 2428, giving an estimated recovery of approximately 30%. Control samples were found to contain minimal PS-NMPs contamination with an average count of ~9 ± 2 (Figure 2C,D). Overall, the 0.1 μm PS-NMPs exhibited greater variability and lower recovery than the 2 μm PS-NMPs, which may be attributed to their significantly smaller size and larger surface area making them more prone to pipetting errors, static attraction to lab materials, and aggregation. While 30% recovery is relatively low, given that this is the first methodology to attempt discrete particle counts for NMPs in this size range from whole tissue samples, as opposed to reporting the mass of NMPs in a sub-sample, this recovery is encouraging and will provide an estimation of counts for NMPs below 1 μm, which has not been previously reported. Additionally, our protocol delivered consistent recovery rates every time; thus, knowing a reliable recovery rate, be it high or low, can be used to calculate and estimate the total particle count in laboratory analyses, which is essential to understanding the bioaccumulation and removal of NMPs in the body. Accounting for particles in this range is critical to understanding NMPs accumulation and health risks. Representative images of two fields of view used for the quantification of 2 and 0.1 μm PS-NMPs are shown in Figure 2A,C, respectively.

### 2.2. Proof-of-Concept Study

**Figure 3:**
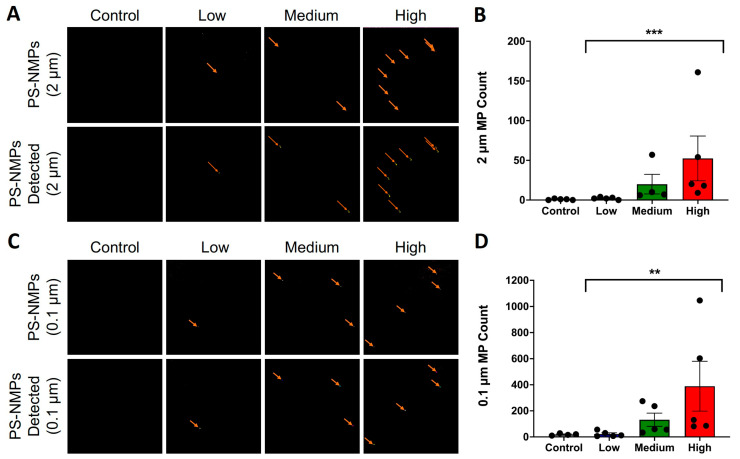
**Quantification of 0.1 and 2 μm PS-NMPs in kidney from PS-NMPs-exposed mice.** (**A**) Representative fluorescence images showing 2 μm PS-NMPs in kidney samples from mice exposed to different concentrations of a 1:1 mixture of 0.1 and 2 μm PS-NMPs. Images shown are before (**upper panels**) and after (**lower panels**) software (ImageJ v2.1.0/1.53c) recognition to demonstrate accuracy, with NMPs denoted with arrows. Field of view = 15 mm^2^. (**B**) Quantification of 2 μm PS-NMPs count in kidneys from PS-NMPs-exposed mice (N = 5 per condition). Significances determined with a Kruskal–Wallis Test with *p* = 0.0009. (**C**) Representative fluorescence images showing 0.1 μm PS-NMPs in kidney samples from mice exposed to different concentrations of a 1:1 mixture of 0.1 and 2 μm PS-NMPs. Images were acquired using an automated spinning-disk confocal system (Opera Phenix) for high-content screening and were automatically analyzed using the Harmony software. The images shown are before (**upper panels**) and after (**lower panels**) spot detection to demonstrate the accuracy of the automated counting, with NMPs denoted with arrows. Field of view = 10,000 μm^2^. (**D**) The quantification of 0.1 μm PS-NMPs count in kidneys from PS-NMPs-exposed mice (N = 5 per condition). Significances determined with Kruskal–Wallis Test with *p* = 0.0030 and denoted with ** *p* < 0.01 and *** *p* < 0.001.

To validate our method, we tested if we could extract, isolate, and quantify PS-NMPs in kidney samples from animals previously exposed [46] to a 1:1 mixture of 2 and 0.1 μm PS-NMPs at low (0.0025 mg/mL), medium (0.025 mg/mL), and high (0.125 mg/mL) concentrations, as compared to control animals receiving no PS-NMPs (Figure 1C). For the control mice, minimal PS-NMPs were detected, with an average of ~1 ± 0.4 for the 2 μm PS-NMPs and ~19 ± 4 for the 0.1 μm PS-NMPs per kidney. In the low-dose group, the average PS-NMP count was similar to that of the controls, with ~2 ± 1 for the 2 μm particles and ~22 ± 9 for the 0.1 μm particles, suggesting that this group may be near or below the limit of detection for this method. Kidneys from the medium- and high-dose groups contained an average of ~20 ± 12 and ~52 ± 28 for the 2 μm PS-NMPs, and ~131 ± 51 and ~389 ± 191 for the 0.1 μm PS-NMPs, respectively (Figure 3A–D). The increase in average PS-NMP count as the dose increased suggests that this method may be useful to detect dose-dependent changes in PS-NMP bioaccumulation (Figure 3B,D). As in the recovery rate calculations, outliers were detected using the ROUT method with a Q value of 5% and were removed from the analysis. It should be noted that one sample in the medium-dose group was determined to be an outlier for the 2 μm PS-NMPs, as it contained 389 NMPs of this size. With a sample size of 5, including such a drastic outlier at present may result in an average that is not truly reflective of the population and result in misleading interpretations of the data. As this methodology will be applied in future studies, larger sample sizes should be used to gain a true understanding of the dose–accumulation relationship in NMPs exposure studies. Full data for the present study will be made available upon request.

## 3. Discussion

In this study, we developed a method to extract, isolate, and quantify mixed size 2 and 0.1 µm PS-NMPs from mammalian tissue and determined the recovery rate of this method to be 85% for 2 µm and 30% for 0.1 µm PS-NMPs. Using the workflow shown in Figure 1, we first conducted a recovery rate calculation in which kidneys were collected from C57BL/6J mice, with a subset serving as controls (Figure 1A) and a second subset spiked at the start of the workflow with 5 µL each of 2.5 µg/mL solutions of 2 and 0.1 µm PS-NMPs to create a 1:1 spiking volume mixture (Figure 1B, denoted SR). Spiking a portion of the samples at the start of the workflow allowed us to mimic an actual sample containing NMPs and determine the amount of recovered NMPs after the entire workflow had been completed. The remaining kidney samples were either left blank (procedural controls) or spiked with our starting PS-NMPs solutions immediately prior to imaging, but after the extraction workflow, to determine the starting quantities of PS-NMPs in our working solutions (Figure 1B, denoted 2 or 0.1 µm SQ). This is a critical step that is often overlooked when in NMPs quantification studies. It is essential to use these SQ samples as a positive control to determine the amount of PS-NMP particles in our spiking volume, without any loss due to the extraction process, to determine recovery rate, which is pivotal in understanding how reflective our reported values are of true NMPs accumulation.

In many previous studies using techniques such as Py-GC/MS, the starting value of NMPs is assumed to be the concentration provided by the manufacturer [74]. While the overall mass concentration may be accurate, the actual particle count in these samples may vary widely, especially as the size of the NMP decreases. This principle is well highlighted in Simon et al., which examined both mass and particle counts of microplastics 10–500 μm in wastewater and found significant variation in particle count for samples with similar mass values [75]. For a 0.1 µm spherical NMP with a density of ~1 g/cm^3^, as used in the present study, the mass of a single particle may be less than 1 femtogram (10^−15^ g). This can make calculating true recovery rates very difficult, since mass-based results are likely to result in overall less variability and higher recoveries. For example, the difference between our SQ and SR samples for the 0.1 µm PS-NMPs appears as only a 30% recovery in terms of particle count, but it is critical to keep in mind that this is a theoretical mass difference of <0.00001 µg, which would appear negligible using Py-GC/MS, and therefore may be miscalculated as a higher recovery than it truly is. This inability to detect such variations and the masking of significant particle loss can make it difficult to compare reported values across studies and be confident that these values accurately reflect total NMPs accumulation.

These are just a few of many factors to consider when comparing our methodology to others. In addition to masking losses through mass-based measurements, many studies do not provide reports of recovery rate [54,55], which can make it difficult to understand how reflective reported results are of the actual sample being processed. On the other hand, some studies have reported recovery rates as high as nearly 100% [76,77,78]; however, it is important to acknowledge that these rates are again based on mass values as opposed to particle count, are typically for larger microplastics rather than nanoplastics, and are often in simpler sample matrices such as water rather than biological tissue. With the field of NMPs research being so vast, it is important to keep these parameters, such as particle size and sample matrix, in mind when selecting a quantification technique or comparing different studies. For example, it is unrealistic to expect to use the same technique to assess large microplastics (>1 mm) in water and nanoplastics in mammalian tissues and achieve similar recovery rates.

A recovery of > 80% for most techniques will deem the method accurate and reliable. While we recognize that 30% recovery of the 0.1 µm NMPs may be deemed low and thus “unsuccessful”, it is important to recognize that we are reporting for the first time a recovery rate for nanoplastic particle counts; hence, it cannot realistically be compared to the higher recovery rates of the larger (e.g., 2 µm) microplastic particle counts. The significant differences in the recovery rates we show here highlight how much the small size and high surface area of nanoplastics contribute to losses during extraction processes, yet these losses are often not factored into reported values. Future studies may try to reduce the number of processing steps, minimize use of materials with high static attraction, and use less aggressive digestion agents as ways to improve the nanoplastic particle recovery rate from tissue.

While there is still a need to continue to improve this recovery rate and the methodology overall, being able to provide a more reliable estimation of counts for NMPs of this size is a huge step forward for NMPs research. This may be especially useful in cases where NMPs accumulation is being compared, such as between a healthy and disease group, for example. A difference of a few hundred 0.1 µm NMPs between groups would be undistinguishable using Py-GC/MS and would be too time consuming to determine from whole tissues via Raman, FTIR, or SEM, but could have significant implications in the correlation between NMPs accumulation and disease outcomes. This method may be applied to detect such differences and allow us to answer long-standing questions about adverse health outcomes connected to NMPs accumulation.

To verify that this methodology could be applied to an actual laboratory setting, we next conducted a proof-of-concept study in which kidneys collected from mice exposed to PS-NMPs from our previous study [46] (Figure 1C) were digested and run through our workflow to determine the bioaccumulation of PS-NMPs. Using these samples, we found that we were able to detect a seemingly dose-dependent accumulation of PS-NMPs (Figure 3). It should be recognized that in order to obtain a true understanding of the dose–response relationship for NMPs exposure, a larger sample size should be used. At present, we have used tissues that were collected from a previous study to test our methodology and verify its applicability; however, we hope to conduct more robust studies to better assess NMPs accumulation in the near future. As mentioned previously, one sample in our medium-dose group had 389 of the 2 µm NMPs and was determined to be an outlier using the ROUT outlier test with Q = 5%. This sample was clearly not representative of the group at present, but a larger sample size may help us better understand this observation as well. This is a pitfall that has commonly affected NMPs research, as there has been no method currently fast and robust enough to fully process large amounts of samples in a reasonable time frame. Techniques such as py-GC/MS, Raman, and FTIR can take many hours, or even up to a day, to analyze a single sample or even a single NMP [79,80]. This has made it very common for many NMPs research groups to either work with a small sample size (typically 3–5) or to sub-sample and extrapolate, which can result in misleading quantifications. By comparison, the total time to image both sizes of PS-NMPs with our methodology is ~3 h per sample without sub-sampling the tissue. This increased speed may allow researchers to process larger amounts of samples and thus obtain more robust measures of NMPs bioaccumulation.

In addition to NMP particle characteristics, there are a few important methodological parameters that should be taken into consideration when adapting this method for future studies. The first is that we utilized 30% KOH as a digestion agent. While polystyrene is quite resistant to such a high concentration of KOH [81,82], not all polymers are, and caution should be used when applying a strong base as a digestion agent, as it may result in NMP degradation. Even within this study, we noticed a moderate decrease in fluorescence intensity between particles in our SQ and SR samples, which may suggest some particle degradation by the digestion process. Studies exploring unknown NMPs from environmental sources may need to adjust KOH concentration or utilize a less aggressive digestion agent to minimize NMP loss. It should be noted, however, that even other seemingly more mild digestion agents, such as Fenton’s reagent, can cause significant particle degradation, especially as the size of the NMP decreases and its surface area to volume ratio increases [83]. Furthermore, we selected nylon filters for its resistance to KOH. It is important to select a filter with the digestion agent and particle size in mind so that the filter is not degraded during the extraction process and efficient separation/isolation of NMPs is achieved [81,84]. Alternative materials, such as metal and silica, may be considered for filters to minimize sample contamination with synthetic fibers, although we found that there was limited commercial availability of filters with sub-micron pore sizes in these materials. Additionally, we used PS-NMPs that already had a fluorescent label embedded, which aided in visualization. Should this technique be applied to environmental samples or non-fluorescent particles, additional steps might need to be included, such as staining with Nile red [85] or using an automated spinning-disk confocal with a higher magnification to visualize such small NMPs. Given the highly varied nature of NMPs research, it is also critical that future studies using this methodology continue to conduct recovery rate studies to ensure accurate and comparable NMPs reporting, including factors such as the sample matrix, size of NMPs, polymer type, etc., all of which can influence recovery rate.

The field of NMPs research is one that is only continuing to grow in complexity. Globally, plastic production, and therefore plastic pollution, is growing at a nearly exponential rate that has resulted in an ever-growing accumulation of NMPs in the environment. Early research has shown that these NMPs can be ingested, inhaled, and absorbed by organisms of all trophic levels including humans, and exert numerous adverse effects such as inducing inflammation, altering metabolism, and disrupting major organ systems [7,8,9,10,11,12,13,14,15,16,17,18,19]. As such, it has become increasingly critical to assess bioaccumulation and perform health studies in model organisms in order to conduct risk assessments and provide a better understanding of the effects of NMPs exposure. Previous methodologies to assess NMPs accumulation have many benefits, have provided a critical framework for understanding NMPs toxicity, and have been largely successful for quantifying larger NMPs; however, there is still much improvement to be made. In particular, there has been a distinct lack of techniques for whole tissue discrete particle counts of sub-5 µm NMPs. NMPs in this size range are critical to assess, as they may be the most abundant size of NMPs and their exclusion may lead to an underrepresentation of total NMP accumulation. With this in mind, we hope that our newly developed workflow for the extraction, isolation, and quantification of 2 and 0.1 µm PS-NMPs may be modified and applied to a wide range of health studies to better understand outstanding questions of how factors such as sex, age, diet, activity level, and disease states, for example, may impact the accumulation of NMPs and the overall fate of NMPs in the body.

## 4. Conclusions

We set out to develop a continuous workflow for the extraction, isolation, and quantification of mixed 2 and 0.1 µm PS-NMPs from mammalian tissues, and to determine the recovery rate of both sizes by assessing particle counts. Using our methodology, we were able to achieve 85% recovery and 30% recovery for 2 and 0.1 µm PS-NMPs, respectively. We were further able to validate this method using kidney tissue from experimentally exposed mice to detect the dose-dependent bioaccumulation of PS-NMPs. This workflow provides a relatively fast and robust method for quantifying particle counts of mixed micro- and nanoplastics from whole mammalian tissues, which has not previously been achievable, and may be adapted for future studies to better answer questions regarding NMPs bioaccumulation and toxicity.

## 5. Materials and Methods

### 5.1. PS-NMPs Particle Characterization

The diameters and shape of PS-NMPs were further confirmed via scanning electron microscopy (SEM; Figure S1). SEM imaging was performed on a Zeiss Sigma VP field emission scanning electron microscope at 5 kV and either 5× or 50× magnification depending on the size of the PS-NMPs. The stock PS-NMPs as provided by the manufacturer were drop cast onto silicon, dried, and gold-coated to minimize charge effects. ImageJ analysis was subsequently used to assess average diameter, and the general morphology of PS-NMPs was visually examined. To verify the chemical composition of the PS-NMPs, 2 µm PS-NMPs as provided by the manufacturer were scanned using an inVia Qontor Raman microscope (Renishaw, Gloucestershire, UK; Figure S2). Spectra were acquired under 100× magnification using a 785 nm laser, and for one 10 s accumulation. Spectra were processed to remove fluorescence background, perform baseline subtraction, noise filtration, and cosmic ray removal using the WiRE software (v5.4) prior to database matching against the Hawaii Pacific University Center for Marine Debris Research Polymer Kit Reference Library 1.0, with a 80.9% match achieved [86,87]. The 0.1 µm PS-NMPs were below the limit of detection for chemical composition analysis via Raman, but were purchased from the same manufacturer as the 2 µm PS-NMPs.

### 5.2. Experimental Design

Kidney samples for recovery rate calculations (Figure 1A) were collected from 4 to 10-month-old C57BL/6J mice (N = 45) bred in house. Kidney samples for the proof-of-concept study (Figure 1C) were collected from 4-month-old female C57BL/6J mice (N = 20) received from the National Institute of Aging (NIA) aged rodent colony (Charles River Laboratories, Kingston, NY or Raleigh, NC, USA). The NMPs exposure conditions for the proof-of-concept mice were fully described in a previous study [46]. Briefly, mice were divided into four exposure conditions: control (normal drinking water), low (0.0025 mg/mL), medium (0.025 mg/mL), and high (0.125 mg/mL), and received a 1:1 volume mixture of 0.1 and 2 µm fluorescently labeled pristine spherical polystyrene microplastics (PS-NMPs, R100TS/R0200, ThermoFisher Scientific, Waltham, MA, USA) for 3 weeks of exposure via drinking water. The diameters of PS-NMPs were validated by the manufacturer via laser diffraction and all PS-NMPs were rinsed 3 times with deionized water and centrifuged at 21,000× *g* for 1 h and 45 min prior to use to remove trace amounts of surfactant. All mice received water and a standard diet (Teklad Global Soy Protein-Free [Irradiated] type 2920X, Envigo, Indianapolis, IN, USA) ad libitum and were housed with a small hut and tissues for nesting in a ventilated cage, with up to 5 mice per cage. The mice were kept on a 12:12 light–dark cycle at 22 °C ± 1 and 30–70% humidity. Appropriate measures were taken to minimize animal pain and discomfort. The investigation was conducted in accordance with the ethical standards and according to the Declaration of Helsinki and national and international guidelines and has been approved by the authors’ institutional review board.

### 5.3. Recovery Rate Calculations

To begin the workflow, kidney samples (N = 45, Figure 1A) were removed from −80 °C storage and immediately placed in 16 mL borosilicate glass test tubes (Figure 1B, Step 1). A subset of the kidney samples (N = 15) was spiked with 5 µL each of 2.5 µg/mL 2 and 0.1 µm PS-NMPs; all other samples were left blank. These spiked samples are meant to mimic actual samples and undergo the entire extraction workflow (Figure 1B); thus, the PS-NMPs that remain at the end are determined to be the recovered PS-NMPs. These samples will herein be referred to as “spike recoveries” (SRs). All test tubes were covered with a 1-inch square of aluminum foil and given to an outside observer for sample anonymization. This was carried out to avoid researcher bias in NMPs quantification and to help better identify potential cross-contaminations. All samples were then dehydrated for 48 h in a 60 °C oven (Thermo Scientific Heratherm^TM^, Waltham, MA, USA) to remove excess fluids to maintain digestion solution concentration (Figure 1B, Step 2). After dehydration, tissue digestion was achieved by adding 2.5 mL of a 30% potassium hydroxide (KOH, Sigma Aldrich, St. Louis, MO, USA) solution to each test tube (Figure 1B, Step 3). Samples were placed in a 40 °C water bath (Fisher Scientific Isotemp^TM^ 110, Waltham, MA, USA) for 24 h, swirling intermittently to facilitate tissue digestion (Figure 1B, Step 4). Digested samples were then vacuum filtered onto 47 mm 0.8 µm nylon filter membranes (Sterlitech, Auburn, WA, USA) to isolate the 2 µm PS-NMPs onto the filter (Figure 1B, Step 5). Should this workflow be applied to environmental NMPs, alternative filter materials, such as silica, may be considered to avoid sample contamination with nylon fibers. A second glass test tube was affixed to the bottom of the vacuum apparatus to collect 0.1 µm PS-NMPs in the sample filtrate. Minimal rinsing (1–2 mL) of the sample and glass vacuum cup was performed using 30% isopropanol (Sigma Aldrich, St. Louis, MO, USA) to reduce the presence of fatty acids, which may clog filters. Nylon filter membranes (selected for their resistance to KOH) were moved to glass slides treated with a small quantity of Skin-Tac adhesive [88] for imaging and analysis (Figure 1B, Step 6). Immediately prior to imaging, half of the blank sample filters (N = 15) were spiked with 5 µL of the 2.5 µg/mL 2 µm PS-NMPs. By spiking these blank samples after the extraction process had been completed, we may use these samples to quantify the 2 µm PS-NMPs initially present in our spiking volume. These samples will herein be referred to as 2 µm “spike quantifications” (2 µm SQs). Nylon filter membranes were imaged on an automated fluorescence microscope (Olympus BX63, Tokyo, Japan). Images were acquired under 10× magnification using a fluorescence excitation light with wavelength of 480 nm and longpass filter of 505 nm and Z-stacked to capture all material in focus on the filter surface. Counts of 2 µm PS-NMPs from the image were either performed manually by the authors (if PS-NMPs count < 500) or by using CellSens Dimensions Count and Measure function (Olympus/Evident, Tokyo, Japan) (if PS-NMPs count > 500). Automated counts were visually reviewed by the authors for identification quality.

The collected filtrates from all samples were transferred from the glass test tubes to centrifugal filter units with a pore size less than 10 nm (Figure 1B, Step 7) (MilliporeSigma, UFC800396, Burlington, MA, USA) and centrifuged at 2000× *g* for 3 h (Figure 1B, Step 8). Approximately 100–250 µL of the sample remained within the filter unit, and this volume was transferred to a 384-well plate (Revvity, 6057302, Waltham, MA, USA) with a maximum volume of 50 µL per well (Figure 1B, Step 9). Immediately prior to imaging, half of the blank samples (N = 15) were spiked with 5 µL of 2.5 µg/mL 0.1 µm PS-NMPs. By spiking these samples after the extraction process had been completed, we may use these samples to quantify the 0.1 µm PS-NMPs initially in our spiking volume. These samples will herein be referred to as 0.1 µm “spike quantifications” (0.1 µm SQs). The plate was allowed to dry at 37 °C for ~120 h to ensure maximum attachment of MPs to the plate surface (Figure 1B, Step 10). This was carried out to minimize NMPs floating outside the plane of focus during imaging. Once dry, all samples were re-hydrated with 100 µL of DI water (Figure 1B, Step 11) 5 min prior to imaging on the Opera Phenix (Revvity, Waltham, MA, USA) (Figure 1B, Step 12). Re-hydration was required to allow for the auto-focusing of the microscope. All images on the Opera Phenix were collected using the 63× water objective, confocal mode, and Alexa 568 channel. All 0.1 µm MPs counts were determined using the analysis feature on the Harmony software (V4.9, Revvity, Waltham, MA, USA).

### 5.4. Proof-of-Concept Study

Following the calculation of recovery rates, a proof-of-concept study was conducted to determine the applicability of the protocol summarized in Figure 1 in a laboratory setting. Kidney samples were collected from mice exposed to varying concentrations of 0.1 µm and 2 µm PS-NMPs (Figure 1C) [46]. The workflow outlined in Figure 1B was then applied as previously described. Since no samples were spiked in this study, all PS-NMPs counted were attributed solely to the ingestion of PS-NMPs.

### 5.5. NMP Contamination Control

To minimize external NMPs contamination prior to the laboratory processing of samples, the procedures described by Haskell et al. were followed [89]. In summary, all glass and metalware were scrubbed with soap and DI water, rinsed with pure water (Milli-Q^TM^), and air-dried under a laminar flow cabinet (Captair Flow Smart 483-714, Rowley, MA, USA) in a HEPA air-filtered room (Dyson Pure Cool^TM^ TP01, HarbourFront, Singapore). Where possible, all work was conducted under a laminar flow cabinet [90,91]. All glass and metalware requiring repeated use throughout the procedure (glass vacuum cups, tweezers, etc.) were scrubbed with soap and DI water and rinsed with pure water between sample replicates. All reagents were filtered to 0.2 µm prior to use.

### 5.6. Statistical Analysis

Data are presented as mean values (M) with SE and are indicated in the figure legend together with sample size (N). All data collected were analyzed with α level of 0.05 using appropriate software (GraphPad Prism, v.9, San Diego, CA, USA). All outliers were determined using the Prism ROUT outlier test with a false discovery rate of 5% (Q = 5%). Full data will be made available upon request. The specific statistical analyses used were indicated in figure legends with significances denoted in figures as * *p* < 0.05, ** *p* < 0.01, *** *p* < 0.001, **** *p* < 0.0001.

## Data Availability

Data presented in this study are available from the corresponding authors upon reasonable request.

## Supplementary Materials

The following supporting information can be downloaded at: https://www.mdpi.com/article/10.3390/nano15110812/s1; Figure S1: Representative SEM images of 2 and 0.1 µm PS-NMPs; Figure S2: Representative Raman spectrum of 2 µm PS-NMPs.

## Abbreviations

The following abbreviations are used in this manuscript:

AFM	Atomic Force Microscopy
DI	Deionized
FTIR	Fourier Transform Infrared Spectroscopy
KOH	Potassium Hydroxide
NMPs	Nano- and Microplastics
PS-NMPs	Polystyrene Nano- and Microplastics
Py-GC/MS	Pyrolysis–Gas Chromatography/Mass Spectrometry
SEM	Scanning Electron Microscopy
SQ	Spike Quantification
SR	Spike Recovery
