# Supplementary material for: A Workflow for Assessing Particle Counts of Mixed Micro- and Nanoplastics in Exposed Laboratory Animals
Source: Nanomaterials (Basel). 2025 May 28;15(11):812. doi: 10.3390/nano15110812 (PMC12158161; doi:10.3390/nano15110812)
Supplement: Supplementary file 1 [file nanomaterials-15-00812-s001.zip › nanomaterials-3633569-supplementary.pdf]

A **B**

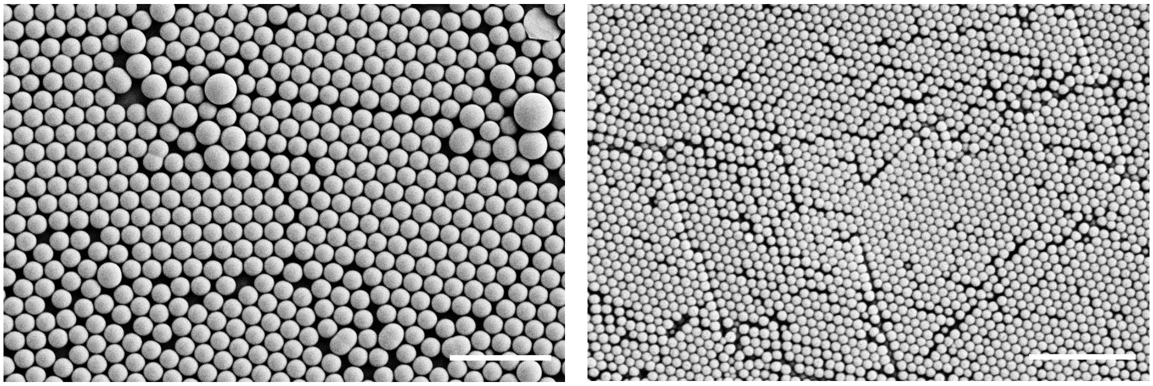

Supplementary Figure 1. Representative SEM Images of 2 and 0.1 µm PS-NMPs. (A) Representative SEM image showing 2 µm PS-NMPs as purchased from the manufacturer. Scale bar = 10 µm. ImageJ analysis determined the average diameter to be 2.09 ± 0.01 µm. **(B)** Representative SEM image showing 0.1 µm PS-NMPs as purchased from the manufacturer. Scale bar = 1000 nm. ImageJ analysis determined the average diameter to be 99.52 ± 2.52 nm.

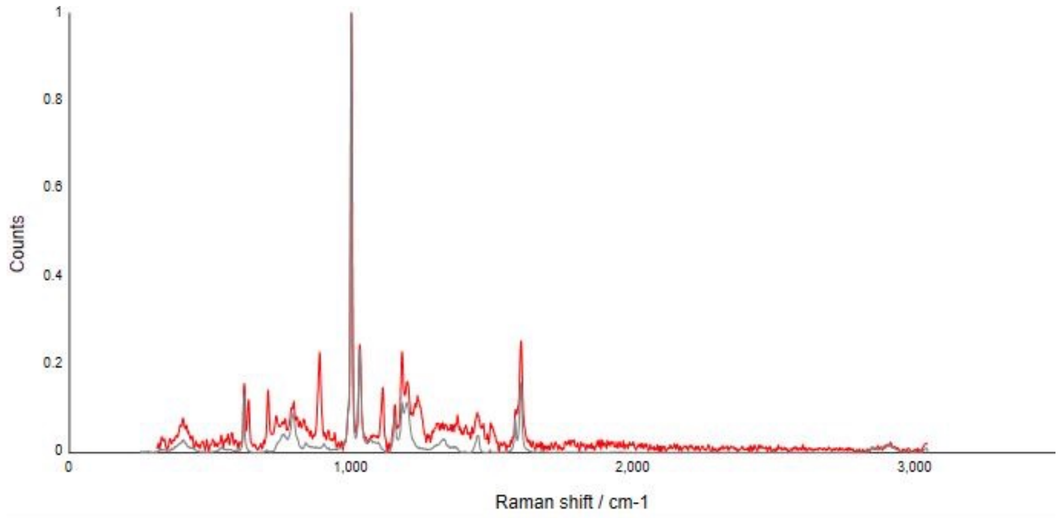

Supplementary Figure 2. Representative Raman Spectrum of 2 µm PS-NMPs. Representative image of Raman spectrum of 2 µm PS-NMPs shown to validate polystyrene composition. 2 um PS-NMPs were analyzed using an inVia Qontor Raman Microscope. Spectra were acquired under 100x magnification using a 785 nm laser for one 10 s accumulation. Spectra were processed to remove fluorescence background, perform baseline subtraction, noise filtration, and cosmic ray removal using the WiRE software (v5.4) prior to database matching against the Hawaii Pacific University Center for Marine Debris Research Polymer Kit Reference Library 1.0, with an 80.9% match achieved.
